# Validation and Reliability of the Slovenian Translation of the Adherence to Exercise for Musculoskeletal Pain Tool (ATEMPT)

**DOI:** 10.2478/sjph-2026-0010

**Published:** 2026-06-01

**Authors:** Suzana Pustivšek, Maja Dakskobler, Andraž Furlan, Tim Kambič

**Affiliations:** National Institute of Public Health, Prevention and Promotion Programmes Management Centre, Trubarjeva cesta 2, 1000 Ljubljana, Slovenia; University of Ljubljana, Faculty of Sport, Department of Medical Sciences in Sport and Exercise, Gortanova ulica 22, 1000 Ljubljana, Slovenia

**Keywords:** Exercise, Adherence, Chronic musculoskeletal pain, Quality of life, Physical activity, vadba, adherenca, kronična mišičnoskeletna bolečina, kakovost življenja, telesna dejavnost

## Abstract

**Introduction:**

Subjective measures for assessing exercise adherence in patients with non-cancer widespread chronic muscle pain (CMP) are limited. Following the recent development of the valid and reliable Adherence To Exercise for Musculoskeletal Pain Tool (ATEMPT), this study aimed to assess the reliability of the Slovenian translation of the questionnaire for measuring exercise adherence in Slovenian patients with CMP.

**Methods:**

This cross-sectional study included 107 patients with CMP (95% female), with a mean (SD) age of 56 (8) years, to assess the reliability and construct validity of the Slovenian version of ATEMPT. Following initial translation into Slovenian by 2 experienced translators and minor adaptation of terminology after discussing with people and an exercise specialist, the questionnaire was administered on 2 occasions, with a median (IQR) of 8 (0) days between assessments.

**Results:**

Overall, test-retest comparisons showed similar scores for each item and for the score. Correlations between test and retest scores for each item and the total score were positive and moderate to high (all 0.533 < r < 0.733, all p < 0.001). Reliability of each item and the total score was significant and ranged from moderate (first, third and fourth items; intraclass correlation coefficients [ICCs] = 0.688–0.700) to good (second and sixth items and overall score; ICC = 0.824–0.852). Construct validity was strong, with all items loading onto a single underlying factor that explained 57% of the variance in ATEMPT scores.

**Conclusions:**

The Slovenian translation of ATEMPT demonstrated moderate to excellent measurement properties and can therefore be used to assess exercise adherence in people with CMP.

## Izvleček

**Uvod:**

Subjektivni kazalniki za ocenjevanje doslednosti izvajanja vadbe pri osebah z razširjeno ne-rakavo kronično mišično-skeletno bolečino (KMSB) so pomanjkljivi. Z nedavnim razvojem veljavnega in zanesljivega vprašalnika Adherence To Exercise for Musculoskeletal Pain Tool (ATEMPT) smo v naši raziskavi želeli oceniti ponovljivost in veljavnost slovenskega prevoda vprašalnika za preverjanje doslednosti izvajanja telesne dejavnosti pri slovenskih bolnikih s KMSB.

**Metode:**

V presečno raziskavo smo vključili 107 bolnikov z KMSB (95 % žensk) s povprečno starostjo 56 (SD, 8) let. Po prvem prevodu v slovenščino, ki sta ga opravila dva neodvisna prevajalca, smo med pacienti preverili razumevanje vprašalnika. Na podlagi povratnih informacij smo naredili minimalne terminološke prilagoditve vprašalnika, nato smo s pomočjo 1-ka spletnega orodja vprašalnik administrirali dvakrat, pri čemer je bil časovni razmik med meritvama 8 (0) dni.

**Rezultati:**

Na splošno so preiskovanci pri posameznih vprašanjih in pri skupni oceni dosegali podobne rezultate med prvo in ponovljeno izpolnitvijo vprašalnika. Korelacije med prvim in drugim merjenjem za posamezna vprašanja ter skupne ocene vprašalnika so bile pozitivne in srednje visoke do visoke (vse p < 0,001). Zanesljivost posameznih vprašanj in skupne ocene vprašalnika je bila statistično značilna in je segala od zmerne (prvo, tretje in četrto vprašanje; intraklasni korelacijski koeficient ICC = 0,688–0,700) do dobre (drugo in šesto vprašanje ter skupni rezultat; ICC = 0,824–0,852). Veljavnost konstrukta je bila odlična, saj so vsa vprašanja ostala znotraj enega faktorja, ki je pojasnil 57 % variance vprašalnika ATEMPT.

**Zaključki:**

Slovenska različica vprašalnika ATEMPT je pokazala zmerno do odlične merske lastnosti in je zato primerna za preverjanje doslednosti izvajanja telesne dejavnosti.

## 1 INTRODUCTION

Chronic musculoskeletal pain (CMP), defined as persistent or recurrent pain condition arising from muscles, joints, or bones lasting for more than 3 months, and Fibromyalgia Syndrome (FMS), a chronic musculoskeletal disorder of unknown aetiology, are highly prevalent conditions (12.7%–33.7%) across sexes and significantly impair quality of life, including work, functional ability and overall health (1,2,3). Consequently, CMP presents a substantial public health burden, accounting for up to 4% of gross domestic product (12 billion euros) in 2016 in developed countries such as Norway, with 80% of these costs attributed to productivity loss due to work absence (4). The greatest burden of CMP has been reported in women and those with more than 3 pain sites (5).

While regular physical activity (PA) is widely recognised as a key, evidence-based component of therapy for managing these conditions, contributing to improvements in pain, physical function, and overall wellbeing, people with CMP often avoid PA (6). Adherence to exercise may vary according to individual factors such as motivation, self-efficacy, and anxiety, as well as intervention-related factors such as exercise difficulty and required time commitment (7). Adherence to prescribed PA and exercise training programmes among people with CMP and FMS often remains suboptimal and may limit the achievement of desired therapeutic outcomes (1, 8,9,10,11).

The assessment of exercise adherence remains a significant challenge in clinical research due to the lack of specific measurement tools and the abundance of inconsistent approaches, which mostly rely on questionnaires, self-report diaries and logbooks (12, 13). In attempt to standardise the evaluation of exercise adherence, novel, valid and reliable measures have been developed, such as the 6-item Exercise Adherence Rating Scale for chronic low back pain (13) and the 6-item Adherence To Exercise for Musculoskeletal Pain Tool (ATEMPT) (14), which include key domains of exercise adherence (communication with professionals; targets; how exercise is prescribed; knowledge and understanding; motivation and support; and psychological approach and attitudes) for people with MSK pain (15). Overall, this robust development demonstrated adequate content and structural validity, good internal consistency and acceptable test-retest reliability, making ATEMPT a feasible measurement tool for further translation and cultural adaptation for use in clinical rehabilitation settings (13, 14). Therefore, the aim of this study was to culturally adapt and validate the Slovenian version of ATEMPT to provide an efficient clinical tool for assessing the association between physical exercise and pain perception in people with CMP and/or FMS.

## 2 METHODS

### 2.1 Study design and sample

**Figure 1.**
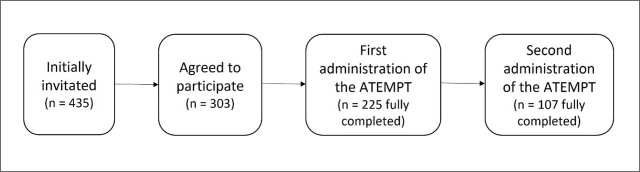
Questionnaire administration flow.

A cross-sectional study was conducted in accordance with the STROBE guidelines (16). Data were collected over 2-week period in February 2025. The online questionnaire was distributed to 435 people who are members of the Slovenian Fibromyalgia Association, which has 10 patient support groups across Slovenia. A total of 303 participants started the questionnaire, and 225 completed it in full. Eight days after the initial administration, those who had completed the questionnaire were invited to complete ATEMPT again. A total of 113 people responded, out of whom 107 questionnaires were valid (Figure 1). The dataset is openly available in the Zenodo open-access repository (17).

For the 107 participants with CMP, 95% were female, with a mean (SD) age of 56 (8) years, and the median (IQR) interval between test and retest was 8 (0) days. Each participant provided informed consent to take part in the study and could withdraw at any time without providing a reason. The study protocol and assessments were approved by the National Medical Ethics Committee of the Republic of Slovenia (ref. no.: 0120-57/2024-2711-10), and the study was conducted in accordance with the Declaration of Helsinki concerning ethical principles for medical research involving human participants.

### 2.2 Translation process and adjustment of ATEMPT

**Figure 2.**
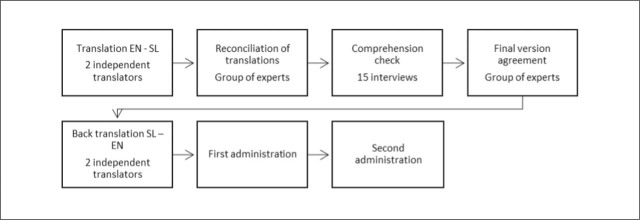
Flowchart of questionnaire validation process.

The Adherence to Exercise for Musculoskeletal Pain Tool (ATEMPT) is a 6-item questionnaire (14) designed to measure exercise adherence in musculoskeletal pain. It addresses the existing limitation of a lack of a standardised, valid, and reliable measurement tool for exercise adherence in clinical practice and research settings. Scored from 6 to 30, the questionnaire functions as a unidimensional measure of exercise adherence, with a change of ≥ 4 points indicating a change beyond measurement error (14). The cross-cultural adaptation of the questionnaire for use in the Slovenian language followed internationally recognised methodological guidelines for the translation and cultural adaptation of self-report measures (18, 19). The main steps are presented in chronological order in Figure 2. Two independent certified translators, each with more than 5 years of professional experience, translated the original English version into Slovenian. Neither translator had a background in exercise science or public health, ensuring a general rather than discipline-specific perspective. The two forward translations were compared and reconciled by a panel of 3 experts in sport science through an online consensus meeting (Zoom platform, Zoom Communication Inc., San Jose, CA, USA). A synthesised version was produced by integrating the most appropriate elements from both translations.

Comprehension and clarity of the pre-final version were assessed through cognitive interviews with 15 participants. Each participant first completed the questionnaire independently and was subsequently asked whether it was understandable and whether they experienced any difficulties during completion. In addition, each item was discussed in detail to identify ambiguous or unclear terms and to propose alternative wording.

The expert panel reviewed the results of the cognitive interviews and approved the final Slovenian version of the questionnaire. Minor modifications were made, involving changes to the wording of the statements:

- “I feel my individual needs were understood when the exercise therapist recommended my exercise” was revised to “I feel my individual needs were considered when the exercise specialist recommended my exercise”.
- “I believe in the exercise that has been recommended to me” was revised to “I believe in the efficacy of exercise that has been recommended to me”.

The term “therapist” was replaced with “specialist” because “exercise specialist” refers broadly to professionals who promote physical activity for health or performance, such as kinesiologists and trainers. In Slovenia, however, the term “exercise therapist” typically refers to a physiotherapist who uses exercise in clinical rehabilitation. The term “exercise specialist” was therefore used to ensure broader applicability across healthcare systems, while primarily referring to healthcare professionals delivering exercise interventions (e.g. physiotherapists and kinesiologists) within healthcare settings.

The final Slovenian version was translated back into English by 2 independent certified translators, each with at least five years of professional experience. Both translators were blinded to the original version to avoid bias. The first administration of the finalised Slovenian version took place in February 2025 via 1KA, an open-source online survey platform (Centre for Social Informatics, Faculty of Social Sciences, University of Ljubljana). A second administration was conducted 8 days later. Data were collected anonymously.

### 2.3 Statistical analysis

Descriptive variables are presented as frequencies and percentages, and continuous numeric variables as mean (standard deviation) or median (first quartile, third quartile) for non-normally distributed variables. Continuous variables were assessed for normality using the Kolmogorov–Smirnov test and the visual inspection of histograms.

Test-retest differences for each item and the overall ATEMPT score were analysed using a paired-samples t-test. Inter-item correlations and correlations between each item and the overall ATEMPT score (i.e. internal consistency) were calculated using the Spearman rank correlation coefficient. Reliability of each item and the overall ATEMPT score was assessed using the intraclass correlation coefficient (ICC [3,1], two-way mixed model for consistency) and interpreted as poor (ICC < 0.50), moderate (0.51 < ICC < 0.75), good (0.71 < ICC < 0.90) and excellent (ICC > 0.91) (20).

Exploratory factor analysis was performed to assess the construct validity of the Slovenian translation of ATEMPT. Prior to analysis, the Kaiser-Meyer-Olkin measure was used to assess sampling adequacy (KMO > 0.5), and Bartlett's test of sphericity (p < 0.05) was used to determine whether correlations between items were sufficiently large for factor analysis. Inter-item correlation analysis was also performed to establish significant associations between all items included in the factor analysis (21). Kaiser-Meyer-Olkin measure of sampling adequacy was interpreted as mediocre (0.5–0.7), good (0.7–0.8), great (0.8–0.9) and superb (> 0.9) (22). The number of factors was determined based on eigenvalues (> 1) and the point of inflexion in the scree plot (20). “Principal component” analysis was used, with varimax rotation method; however, rotation was not applied in the final model, as a single factor was identified (20). All analyses were performed using IBM SPSS version 29 (SPSS Inc. Armonk, NY, USA), with a significance level of p < 0.05 (α = 0.05)

## 3 RESULTS

Respondents reported similar scores for each item and for the total ATEMPT score between test and retest (Table 1), with the exception of a significantly higher score for the first item at retest (mean difference = +0.32 points, p = 0.003).

**Table 1.** Differences between test and retest for each item and the overall ATEMPT score.

**ATEMPT item**		**M**	**SD**	**SEM**	**d**	**t**	**p**
**I feel my individual needs were considered when the exercise specialist recommended my exercise (Item 1)**	Test	3.29	1.24	0.12	−0.318	−3.001	0.003
	Retest	3.61	1.00	0.10			
**I am doing enough exercise to produce a positive change in my musculoskeletal pain (Item 2)**	Test	3.33	1.02	0.10	−0.075	−1.090	0.278
	Retest	3.40	0.96	0.09			
**I am doing my exercise as instructed (Item 3)**	Test	3.71	0.92	0.09	−0.028	−0.324	0.747
	Retest	3.74	0.95	0.09			
**I understand how my exercises will help with my musculoskeletal pain (Item 4)**	Test	3.80	1.07	0.10	−0.15	−1.507	0.135
	Retest	3.95	1.07	0.10			
**I understand the consequences of not doing my exercise (Item 5)**	Test	4.02	1.06	0.10	−0.037	−0.370	0.356
	Retest	4.06	1.05	0.10			
**I believe in the efficacy of exercise that has been recommended to me (Item 6)**	Test	3.80	1.05	0.10	0.009	0.120	0.452
	Retest	3.79	1.03	0.10			
**Overall score**	Test	21.95	4.78	0.46	−0.598	−1.753	0.082
	Retest	22.55	5.03	0.49			

Legend: n = 107; d = mean difference; M = mean; SD = standard deviation; SEM = standard error of the mean; t = test statistic

Correlations between test and retest scores for each item and the total ATEMPT score were all positive, moderate to high, and statistically significant (all p < 0.001, Table 2). Correlations between each item and the overall ATEMPT score were of a similar magnitude.

**Table 2.** Correlations between test and retest scores for each item and correlations with the overall ATEMPT score.

**ATEMPT item**		**Same item**	**Overall score**	
		**Retest**	**Test**	**Retest**
**I feel my individual needs were considered when the exercise specialist recommended my exercise (Item 1)**	Spearman's rho	0.542	0.693	0.760
	p (Spearman's rho)	0.000	0.000	0.000
**I am doing enough exercise to produce a positive change in my musculoskeletal pain (Item 2)**	Spearman's rho	0.771	0.658	0.720
	p (Spearman's rho)	0.000	0.000	0.000
**I am doing my exercise as instructed (Item 3)**	Spearman's rho	0.533	0.555	0.634
	p (Spearman's rho)	0.000	0.000	0.000
**I understand how my exercises will help with my musculoskeletal pain (Item 4)**	Spearman's rho	0.548	0.787	0.793
	p (Spearman's rho)	0.000	0.000	0.000
**I understand the consequences of not doing my exercise (Item 5)**	Spearman's rho	0.545	0.694	0.831
	p (Spearman's rho)	0.000	0.000	0.000
**I believe in the efficacy of exercise that has been recommended to me (Item 6)**	Spearman's rho	0.706	0.745	0.867
	p (Spearman's rho)	0.000	0.000	0.000
**Overall score**	Spearman's rho	0.730	Na	Na
	p (Spearman's rho)	0.000		

Legend: n = 107; rho = Spearman's correlation coefficient; Na = not applicable

Table 3 presents the test-retest reliability for each item question and the overall ATEMPT score. Reliability was statistically significant and ranged from moderate for the first (ICC = 0.688), third (ICC = 0.701) and fourth items (ICC = 0.700) to good for the second (ICC = 0.852), sixth (ICC = 0.824) and overall score (ICC = 0.852).

**Table 3.** Test-retest reliability for each item and the overall ATEMPT score.

**ATEMPT item**		**95% CI of ICC**			
	**ICC**	**lower bound**	**upper bound**	**F**	**p**
**I feel my individual needs were considered when the exercise specialist recommended my exercise (Item 1)**	0.688	0.543	0.787	3.207	0.000
**I am doing enough exercise to produce a positive change in my musculoskeletal pain (Item 2)**	0.852	0.783	0.899	6.763	0.000
**I am doing my exercise as instructed (Item 3)**	0.701	0.562	0.796	3.350	0.000
**I understand how my exercises will help with my musculoskeletal pain (Item 4)**	0.700	0.56	0.795	3.330	0.000
**I understand the consequences of not doing my exercise (Item 5)**	0.678	0.527	0.78	3.102	0.000
**I believe in the efficacy of exercise that has been recommended to me (Item 6)**	0.824	0.742	0.88	5.678	0.000
**Overall score**	0.852	0.782	0.899	6.737	0.000

Legend: CI = confidence interval; ICC = intraclass correlation coefficient, F = test statistic

Table 4 presents the findings of the exploratory factor analysis (EFA). Prior to the analysis, the Kaiser-Meyer Olkin (KMO) measure indicated very good sampling adequacy (KMO = 0.839), and Bartlett's test of sphericity was statistically significant (p < 0.001). A single factor was identified (eigenvalue = 3.433), explaining 57.21% of the variance in ATEMPT scores. Factor loadings were highest for the fourth item (loading = 0.877) and lowest for the second item (loading = 0.605).

**Table 4.** Exploratory factor analysis.

**ATEMPT item**	**Component 1**
	**Factor loading**
**I feel my individual needs were considered when the exercise specialist recommended my exercise (Item 1)**	0.662
**I am doing enough exercise to produce a positive change in my musculoskeletal pain (Item 2)**	0.605
**I am doing my exercise as instructed (Item 3)**	0.706
**I understand how my exercises will help with my musculoskeletal pain (Item 4)**	0.877
**I understand the consequences of not doing my exercise (Item 5)**	0.814
**I believe in the efficacy of exercise that has been recommended to me (Item 6)**	0.836
**Eigenvalue**	3.433
**% variance**	57.21

## 4 DISCUSSION

This study is the first to translate and validate the recently developed questionnaire for exercise adherence in people with CMP into a foreign language for use outside English-speaking countries (23). The Slovenian translation of the 6-item ATEMPT demonstrated strong construct validity and moderate to good reliability.

The assessment of structural validity of the Slovenian ATEMPT, employing exploratory factor analysis, supported a unidimensional (single-factor) solution as the best fit, explaining 57.21% of the questionnaire's total variance. The questionnaire maintained a comparable level of reliability despite minor linguistic adaptations of certain terms. This finding is consistent with the original study that developed ATEMPT (14), which also identified a single-factor structure through EFA. Despite the statistical unidimensionality, it is important to emphasise that the items included in ATEMPT were selected to represent the 6 conceptual domains of exercise adherence (communication with professionals; goals; exercise prescription; education; motivation and support; and psychological approach and attitudes) previously identified by relevant stakeholders, including people with CMP, physiotherapists and researchers (14, 24). This supports strong construct validity and is consistent with public health frameworks that conceptualise adherence as a multifactorial behavioural construct influenced by individual, interpersonal, and system-level determinants. The Slovenian translation of ATEMPT demonstrated good test-retest reliability for the overall score (ICC = 0.852), consistent with the original version (ICC = 0.85) (14), indicating that it provides stable measures of exercise adherence over time. Variability at the item level likely reflects expected short-term fluctuations in behaviour, pain, and self-perception in people with CMP and fibromyalgia (25), while the stability of the overall score suggests that ATEMPT captures a robust underlying adherence construct. This pattern is consistent with previous findings in behavioural measurement, where short-term intra-individual variability in specific behaviours does not necessarily undermine the validity of the composite score (26). Such measurement characteristics are particularly relevant for public health monitoring, where reliable yet sensitive instruments are required to track behavioural outcomes over time and across interventions. In terms of internal consistency, the study reported moderate to high and statistically significant Spearman rank correlations between individual items and the overall ATEMPT score.

From a public health perspective, the availability of a validated adherence tool addresses a long-standing gap in chronic pain management. Prior systematic reviews have consistently highlighted the lack of standardised, valid, and reliable tools for measuring adherence to exercise training in people with CMP (14, 23, 27). In this study, exercise refers to structured exercise interventions prescribed within healthcare settings for the management of chronic musculoskeletal pain and fibromyalgia. Existing measures often lack a clear, consistently applied definition of adherence or were not specifically developed for this patient population, leading to measurement inconsistencies across research and weakening the evidence base for policy and guideline development (14, 23). In contrast, ATEMPT was purposefully developed for this population, supporting its relevance for evaluating adherence-focused interventions at scale and supporting evidence-informed decision making (14).

The availability of a validated Slovenian version of ATEMPT represents a significant advancement for both clinical practice and research within Slovenia. Clinicians (e.g. kinesiologists, physiotherapists, psychologists) can use this tool to monitor adherence to physical activity recommendations during treatment. A decrease in ATEMPT scores may indicate reduced adherence, prompting clinicians to implement adherence-enhancing strategies such as goal setting, personalised feedback, or barrier identification and problem-solving (8, 14). This may support the tailoring of treatment plans and improve outcomes by helping to determine whether limited progress is related to low engagement or ineffective treatment, thereby enhancing the cost-effectiveness of rehabilitation programmes (3, 28,29,30).

CMP is a leading cause of absenteeism and early retirement in Slovenia, particularly in physically demanding occupations (31). The benefits of adherence to exercise may extend from improvements in pain and physical function at the individual level to reductions in indirect costs related to productivity loss and long-term disability (6, 32). In this context, ATEMPT can serve as a practical tool for integrating adherence monitoring into routine care, workplace health promotion programmes, and community-based rehabilitation initiatives, supporting a shift towards more sustainable, prevention-oriented pain management. To provide better healthcare for people with CMP and FMS, accurate measurement of physical activity and perceived pain is essential. A validated questionnaire such as ATEMPT enables clinicians to monitor progress, tailor treatment, and improve outcomes. In research, it provides a consistent approach to evaluating interventions and adherence strategies. Aggregated ATEMPT data can support the evaluation of national rehabilitation programmes, inform resource allocation, and contribute to the surveillance of behavioural outcomes relevant to chronic disease prevention and healthy ageing, which are priorities within public health policy frameworks (33, 34). Despite its strengths, the Slovenian validation study has some limitations. No information was available on participants' clinical characteristics, as reported in similar validation studies. As all participants were recruited through the Slovenian Fibromyalgia Association, they are likely to have shared similar health-related characteristics. Despite the relatively small sample, which is comparable to the original study that included 112 participants, the questionnaire demonstrated good internal reliability, even in the presence of potential daily and weekly fluctuations in pain that may affect responses across administrations and are common in people with fibromyalgia. Furthermore, the study population was predominantly female (95.3%), which limits the generalisability of these findings to men with CMP or FMS. In the original validation of ATEMPT, men were better represented (33.6%) (14). Additionally, exploratory factor analysis supported a single-factor solution, the ATEMPT items conceptually reflect several domains of exercise adherence. Therefore, the possibility of latent multidimensionality cannot be excluded. Further research should include enrolling larger, more diverse samples of participants to identify distinct phenotypes and tailor exercise programmes through a multidisciplinary approach to enhance adherence, as well as translating the questionnaire into other languages and comparing results across different chronic pain populations.

## 5 CONCLUSION

The Slovenian version of ATEMPT represents a valuable tool for assessing exercise adherence in people with CMP and FMS. It demonstrates adequate content and structural validity, acceptable internal consistency, and good test-retest reliability. However, future research is needed to establish its construct validity and responsiveness across more diverse populations within the Slovenian context, and to confirm its full utility in both clinical practice and research settings.
